# Direct wire pacing during measurement of fractional flow reserve: A randomized proof-of-concept noninferiority crossover trial

**DOI:** 10.3389/fcvm.2023.1137309

**Published:** 2023-10-23

**Authors:** Benjamin Faurie, Angela Acheampong, Mohamed Abdellaoui, Ilona Dessus, Jacques Monsegu, Jérôme Wintzer-Wehekind

**Affiliations:** ^1^ Institut Cardiovasculaire de Grenoble, Grenoble, France; ^2^ Université Libre de Bruxelles, Bruxelles, Belgium; ^3^ Université Grenoble-Alpes, CHU Grenoble-Alpes, Grenoble, France

**Keywords:** innovation, FFR = fractional flow reserve, direct wire pacing, adenosine, cardiac pacing

## Abstract

**Background:**

Adenosine administration for fractional flow reserve (FFR) measurement may induce heart pauses.

**Aims:**

To assess the accuracy and tolerability of direct wire pacing (DWP) during measurement of FFR.

**Methods:**

Adults with at least one intermediate coronary artery stenosis (40%–80%) were consecutively enrolled between June 2021 and February 2022 in this randomized, noninferiority, crossover trial (NCT04970082) carried out in France. DWP was applied (DWP) or not (standard method) through the pressure guidewire used for FFR measurement during adenosine-induced maximal hyperaemia. Subjects were randomly assigned to the allocation sequence (DWP first or standard first). A 2-minute washout period was observed between the two FFR measurements performed for each stenosis. The primary endpoint was the reproducibility of FFR measurements between methods.

**Results:**

A total of 150 focal lesions, presented by 94 subjects, were randomized (ratio: 1:1). The FFR values obtained with each method were nearly identical (*R* = 0.98, *p* = 0.005). The mean FFR difference of 0.00054 (95% confidence interval: 0.004 to 0.003) showed the noninferiority of FFR measurement with DWP vs. that with the standard method. Higher levels of chest discomfort were reported with DWP than with the standard method (0.61 ± 0.84 vs. 1.05 ± 0.89, *p* < 0.001), and a correlation was observed between the electrical sensations reported with DWP and chest discomfort (*p* < 0.001). Pauses (*n* = 20/148 lesions) were observed with the standard method, but did not correlate with chest discomfort (*p* = 0.21). No pauses were observed with DWP.

**Conclusions:**

DWP during FFR measurement resulted in accurate and reproducible FFR values, and eliminated the pauses induced by adenosine.

## Introduction

Accurate assessment of the severity and ischaemic nature of coronary lesions is essential for optimizing patient management and determining the most appropriate therapy. Coronary angiography remains the gold standard for evaluating the topography and morphological characteristics of these lesions, and for assessing their consequences on myocardial perfusion. However, the results obtained by coronary angiography vary according to the operator, and this technique alone does not allow conclusions to be made about the ischaemic nature of stenoses classified angiographically as “intermediate” (i.e., around 40% to 80% coronary stenosis).

The determination of translesional pressure differences by fractional flow reserve (FFR) measurement allows separate analysis of each artery or segment, thus avoiding masking of one ischaemic area by another, more severe, ischaemic zone (1–3). Therefore, in cases when angiography alone provides insufficient information to allow definitive characterization of the lesion, FFR measurement enables specific assessment of a potential decrease in distal coronary flow due to coronary stenosis.

FFR is easily measured during routine coronary angiography using a pressure wire to calculate the ratio of coronary pressure distal to a stenosis or diseased segment to aortic pressure under conditions of maximum myocardial hyperaemia (2, 4). This technique has become the reference standard for indexing the haemodynamic significance of coronary artery lesions, providing guidance for revascularization in case of intermediate coronary artery lesions (5–7). Indeed, FFR measurement has been included in the European cardiology guidelines on myocardial revascularization since 2010 (8). However, the maximal hyperaemia required for FFR measurement is usually induced by the intracoronary administration of adenosine (9), which is associated with well documented and relatively frequent side effects. Hypotension, bronchospasm, complete atrioventricular block, arrhythmias, and sinus pause or sinus arrest are a few of the well-known and somewhat common adverse effects of intracoronary adenosine. Although these serious side effects are transient due to the short half-life of adenosine, bradycardia and cardiac pauses occurring during the procedure can cause vagal discomfort in patients. Moreover, questions have been raised concerning the validity of FFR measurements taken during or just after a severe cardiac pause or coughing effort (10). As a consequence, nonhyperaemic, adenosine-independent, methods for assessing stenosis severity have been developed, such as the resting distal coronary pressure to aortic pressure (Pd/Pa) ratio and the instantaneous wave-free ratio (iwFR) (11, 12). Although these methods have been validated, discrepancies between these nonhyperaemic indices and FFR measurements have been reported (13), with misclassification of stenosis severity in around 10%–20% of cases, indicating that these methods should be used with caution in decision-making algorithms (14).

As an alternative to these nonhyperaemic approaches, the use of direct wire pacing (DWP) during FFR could potentially overcome the drawbacks associated with adenosine-induced bradyarrhythmia. The method of direct heart stimulation with a metallic intracoronary guidewire attached to an external pacemaker was first approved in a study conducted nearly 40 years ago (15), and has recently been shown to be safe and effective in the context of transcatheter aortic valve implantation (TAVI) (16–20) and balloon aortic valvuloplasty (21). We hypothesized that DWP through the FFR-metallic guidewire would prevent adenosine-induced bradycardia during FFR measurement, without the precision or validity of the measurement being compromised by the electrical current.

The aim of this study was to determine whether using DWP during the measurement of FFR would be noninferior to the standard method for obtaining accurate FFR values in subjects with intermediate coronary artery stenosis, while allowing the drawbacks associated with the use of adenosine to be eliminated.

## Methods

### Study design and setting

This randomized, noninferiority, crossover, proof of concept trial (NCT04970082) involved conducting two sets of FFR measurements (one using the standard procedure and one using DWP), in a randomized intervention sequence, on a series of intermediate coronary artery stenoses.

The study was carried out in the catheterization laboratory of the *Institut Cardiovasculaire de Grenoble*, Grenoble, France. In accordance with French law, the study protocol received approval from the Ethics Committee CPP (*Comité de Protection des Personnes)* Ile de France VIII, No. 21 05 39, and the French Health Authority (*Agence Nationale de sécurité du médicament et des produits de santé*, ANSM), No. 4482239. The study complied with the reference methodology MR-001 issued by the French data protection agency (*Commission Nationale de l'Informatique et des Libertés, CNIL*), and was conducted in accordance with the Declaration of Helsinki as modified in Fortaleza (2013), and the recommendations on Good Clinical Practice (ICH E6, ISO 14155:2011).

### Subject recruitment and randomization

All subjects identified through routine clinical evaluation as requiring a pressure wire assessment of coronary artery stenosis(es) were considered for participation in the study. Only subjects ≥18 years old, with at least one intermediate coronary artery stenosis (40%–80%) in a non-infarct related artery, who were able to understand the nature of the study and provide informed consent to undergo the diagnostic or interventional coronary procedures, and who had social security coverage were eligible for inclusion. Subjects with a known allergy to adenosine or any of its excipients, those with a permanent pacemaker, those with atrial fibrillation, those with technically inaccessible stenosis(es), those who were under judicial protection, tutorship or curatorship, and those who were participating in another interventional clinical trial were excluded. Pregnant or breastfeeding women were also excluded.

All subjects gave their written consent before inclusion, after having received detailed oral and written information about the study.

### Study procedures

As per routine practice, each subject underwent a medical examination during which the following data were collected: age, height, weight, heart rate, blood pressure, angina status and medical history. An electrocardiogram (ECG) was also performed to identify conduction disorders or arrythmia. Coronary angiography was then performed using standard techniques. Each intermediate coronary artery stenosis (40%–80%) located in a non-infarct-related artery of a subject who fulfilled the study criteria was consecutively included in the study. The first lesion was randomly assigned to the FFR measurement with standard procedure first followed by FFR measurement during DWP, then the investigators alternated lesions' allocation in the groups. In other words, the second lesion was allocated to the FFR measurement during DWP first followed by the FFR measurement with standard method.

Subjects acted as their own control and were not informed of the allocation sequence prior to the intervention.

**Figure 1:**
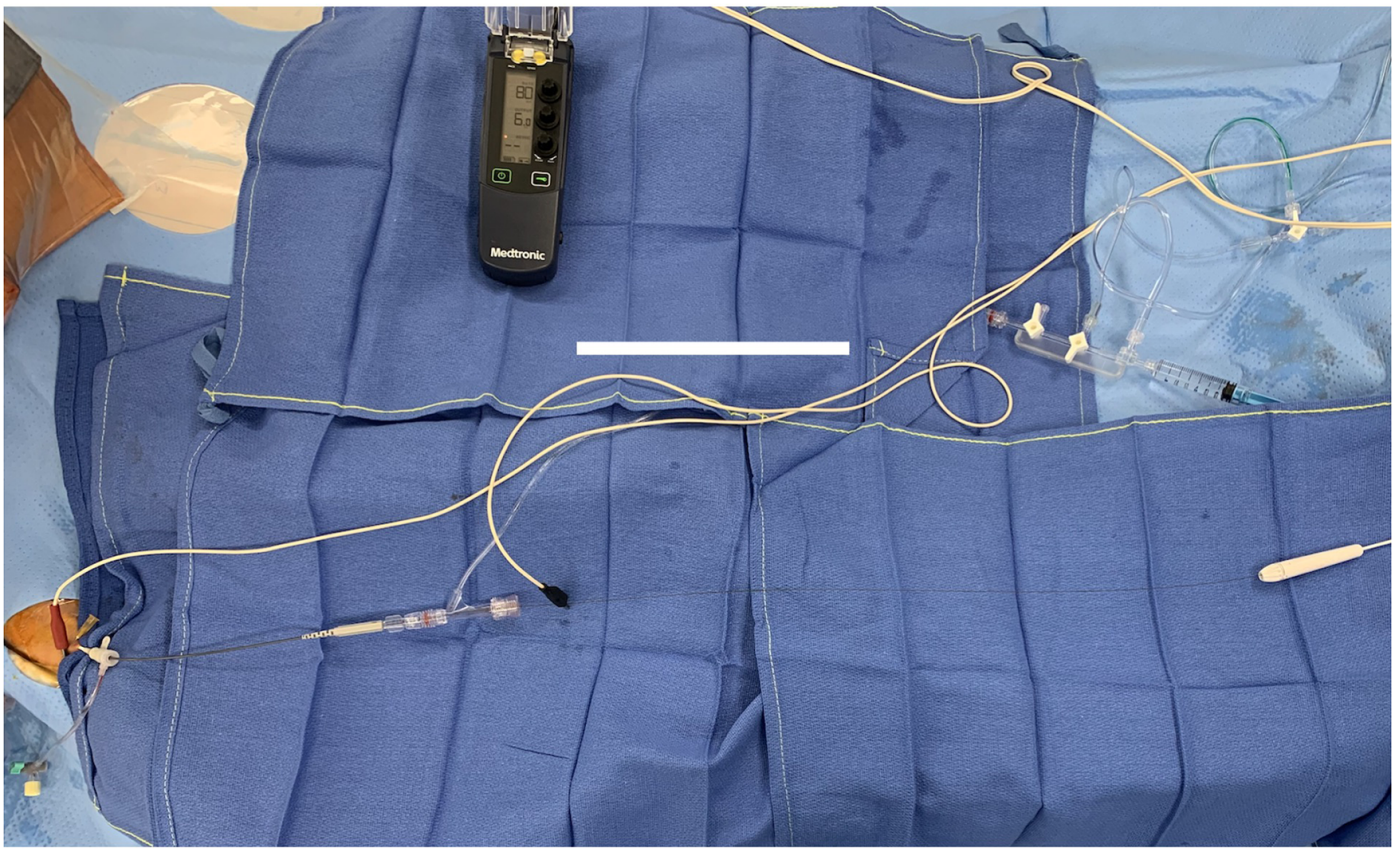
Photo of the material used for FFR measurement during DWP.

In all cases, the FFR was measured by one of the investigators (BF, MA, JM and JWW). For the standard method, a pressure guidewire (optical fibre: OptoWire™, OpSens Medical, Québec, Canada) was inserted into the coronary artery and measurements were obtained at maximal hyperaemia induced by an intracoronary bolus of 200 µg (20 ml) adenosine into the left anterior descending coronary artery (LAD) or 150 µg–200 µg (15 ml–20 ml) into the right coronary artery (RCA). For FFR measurements performed with DWP, at the time of the intracoronary adenosine bolus administration, the heart was stimulated to maximum 10 beats per minute (bpm) above the resting heart rate registered just after positioning the FFR guidewire distally to the lesion. The electrical current was transmitted to the heart through the FFR-metallic pressure guidewire. Two alligator clips were used, the cathode of an external pacemaker was connected to the outer end of the pressure wire and the anode was connected to the skin at the insertion site of the guiding catheter for electrical insulation (Figure 1). A constant current external Pacemaker (Medtronic 53401) was set to asynchronous mode (VOO) starting from 15 mA and the threshold setting was done by decreasing intensity one mA by one mA. Then stimulation intensity was set between two and tree fold the threshold. Although the half-life time of adenosine is very short (below 10 s), a washout period of two minutes was observed between the two FFR measurements in order to eliminate any “carry over effect”.

Haemodynamic and ECG monitoring were performed throughout the whole duration of FFR measurement to record the occurrence of possible bradycardia and pauses (time: < or >3 s).

### Endpoints and outcome measures

The primary endpoint was the reproducibility of the FFR measurement, evaluated by analysis of the differences between FFR values obtained with the two methods (standard and with DWP) for the same lesions. The secondary endpoints included (1) the safety of the two methods assessed by the number of procedures during which participants experienced adverse events; (2) the tolerance of both methods assessed by asking subjects to grade verbally any symptoms of chest discomfort using a 4-point numeric rating scale (NRS: 0, feeling comfortable; 1, slight discomfort; 2, moderate discomfort; and 3, severe discomfort) experienced during each FFR measurement procedure. Immediately after the FFR measurements carried out during DWP, subjects were also asked to grade verbally any electrical sensations they experienced using another NRS ranging from 0, no pain to 10, unbearable pain. Pressure curves and the angiographic degree of stenosis were recorded for each FFR measurement. Details of any adverse events were also collected throughout the study.

### Sample size determination

A 0.02–0.03 difference between two FFR measurements on the same lesion, also called “bias”, is commonly considered acceptable (22–25). In our cardiovascular institute, the difference between two FFR measurements performed in the same lesion with the standard method in 150 lesions (100 subjects) was 0.0194 [99% confidence interval (CI): 0.0151–0.0237]. Therefore, we planned to include 150 lesions in the study in order to use this cut-off value as a noninferiority criterion to determine whether using DWP during the measurement of FFR was noninferior to the standard method.

### Statistical analysis

Quantitative variables were expressed as means and standard deviations (SDs). Qualitative variables were expressed as absolute and relative frequencies, and exact binomial 95% CIs for proportions were calculated when relevant. Missing data were not replaced. For each lesion, the difference between FFR values was calculated as the FFR obtained with the standard method minus the FFR obtained with DWP under maximal hyperaemia. DWP use was considered noninferior to the standard method if the difference between FFR values was not superior to the noninferiority criterion defined above. Baseline characteristics and study outcomes were compared between groups using the *t*-test for means and Chi square test for frequencies. Concordance between the paired FFR values obtained with the standard method and the DWP method was assessed using linear regression analysis (with calculation of the Pearson's correlation coefficient; *R*) and Bland-Altman analysis (with calculation of the mean difference and the 95% CI). Correlation between heart pauses and chest discomfort was assessed with the Spearman correlation coefficient (*ρ*). Chest discomfort was compared between standard and DWP groups using the one-sample *t*-test. The relationship between pauses and tolerability was assessed using the Chi square test, and the relationship between electrical sensation and tolerability was assessed using the Welch test.

Statistical analyses were performed using IBM® SPSS Statistics for Windows (IBM Corporation, Armonk, NY, USA). Statistical significance was set at *p* < 0.05.

## Results

A total of 94 subjects aged 68 ± 11 years (mean ± SD) with 150 focal lesions were prospectively enrolled between June 2021 and February 2022. Most were male (85.1%), with a history of hypertension (69.1%) and/or dyslipidaemia (47.9%). Silent ischaemia (41.5%) and stable angina (39.4%) were the main indications for angiography and FFR measurement. Lesions were located in the left anterior descending coronary artery (LAD) in 50.7% of cases. Around half of the subjects (53.2%) had only one lesion, 33 (35.1%) subjects had two lesions, 10 subjects (10.6%) had three lesions, and one subject (1.1%) had four lesions. No statistical differences in baseline characteristics were observed between lesions assigned to the two groups (Table 1).

**Table 1:** Baseline characteristics.

Characteristics	Standard first	DWP first	*p*-value
	(*N* = 75)	(*N* = 75)	
Age (years), mean ± SD (*m* = 1)	68 ± 11	68 ± 11	*0*.*85*
Gender, *n* (%) (*m* = 1)			*0*.*60*
Female	9 (12.0)	9 (12.2)	
Male	66 (88.0)	65 (87.8)	
BMI, mean ± SD	26.8 ± 4.2	26.7 ± 4.5	*0*.*88*
Smoking status, *n* (%)			
Current smoker	22 (29.3)	19 (25.3)	*0*.*58*
Former smoker	14 (18.7)	16 (21.3)	*0*.*68*
History, *n* (%)			
Hypertension	51 (68.0)	49 (65.3)	*0*.*73*
Dyslipidaemia	35 (46.7)	35 (46.7)	*1*
Type II diabetes	22 (29.3)	22 (29.3)	*1*
Type I diabetes	1 (1.3)	0 (0.0)	*0*.*32*
Renal insufficiency	0 (0.0)	0 (0.0)	*NA*
Family history of CAD	19 (25.3)	20 (26.7)	*0*.*85*
Indication, *n* (%)			* *
Silent ischaemia	27 (36.0)	26 (34.7)	*0*.*86*
Stable angina	33 (44.0)	34 (45.3)	*0*.*87*
Unstable angina	2 (2.7)	2 (2.7)	*1*
NSTEMI	10 (13.3)	9 (12.0)	*0*.*81*
STEMI	3 (4.0)	4 (5.3)	*0*.*7*
Cardiac imaging available, *n* (%)	75 (100.0)	75 (100.0)	*1*
Echocardiography	75 (100.0)	75 (100.0)	*1*
Stress echocardiography	8 (10.7)	8 (10.7)	*1*
Myocardial scintigraphy	11 (14.7)	11 (14.7)	*1*
Cardiac CT	6 (8.0)	4 (5.3)	*0*.*51*
Cardiac MRI	0 (0.0)	0 (0.0)	*NA*
Stress test, *n* (%)	7 (9.3)	10 (13.3)	*0*.*44*
Trunk, *n* (%) (*m* = 2)			*0*.*29*
Left	55 (73.3)	57 (78.1)	* *
Right	20 (26.7)	16 (21.9)	* *
Target coronary artery, *n* (%) (*m* = 2)			*0*.*48*
LAD	37 (49.3)	39 (52.0)	
RCA	20 (26.7)	16 (21.3)	
Cx	14 (18.7)	14 (18.7)	
Intermediate	1 (1.3)	3 (4.0)	
CAT	3 (4.0)	1 (1.3)	

BMI, body mass index; CAT, common arterial trunk; CAD, coronary artery disease; CT, computed tomography; Cx, circumflex artery; LAD, left anterior descending artery; m, missing data; MRI, magnetic resonance imaging; NA, not applicable; NSTEMI, non-ST-elevation myocardial infarction; RCA, right coronary artery; SD, standard deviation; STEMI, ST-elevation myocardial infarction.

**Figure 2:**
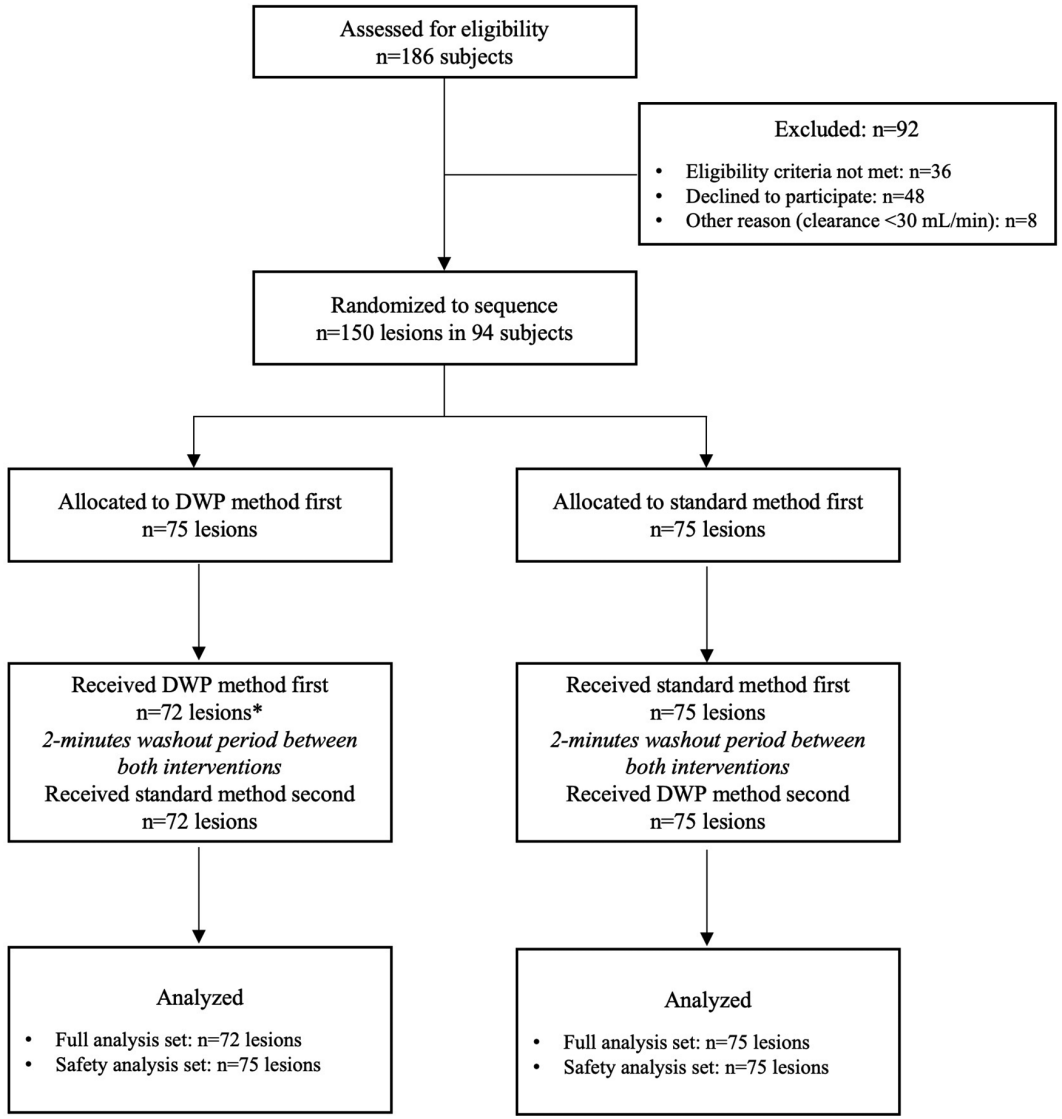
CONSORT flow diagram for crossover trials DWP: direct wire pacing *permanent pacemaker (*n* = 1), coronary occlusive dissection (*n* = 1), and damaged pressure wire (*n* = 1).

No subject withdrew prematurely from the study (Figure 2). FFR measurement could not be performed in three lesions due to a permanent pacemaker (*n* = 1), coronary occlusive dissection (*n* = 1), and a damaged pressure wire (*n* = 1). Procedures were performed in the correct randomized order in all the remaining cases. No differences in the study outcomes were observed between lesions assigned to the two groups (Table 2).

**Table 2:** Study outcomes.

	Standard first (*N* = 75)	DWP first (*N* = 72)	*p*-value
Resting heart rate (bpm), mean ± SD	72 ± 11	73 ± 9	*0*.*91*
Stimulated heart rate (bpm), mean ± SD	78 ± 12	79 ± 11	*0*.*59*
Pacing threshold (mA), mean ± SD	5 ± 3	5 ± 3	*0*.*90*
FFR with the standard method, mean ± SD	0.81 ± 0.11	0.82 ± 0.09	*0*.*46*
FFR with DWP, mean ± SD	0.80 ± 0.12	0.82 ± 0.09	*0*.*26*
Chest discomfort after the standard method, mean ± SD	0.65 ± 0.81	0.58 ± 0.87	*0*.*57*
Chest discomfort after DWP, mean ± SD	1.05 ± 0.82	1.04 ± 0.97	*0*.*94*
Electrical sensation after DWP, mean ± SD	2.96 ± 2.49	2.79 ± 2.82	*0*.*70*
Pauses observed with the standard method, *n* (%)			*0*.*10*
No pause	65 (86.7)	63 (86.3)	
Pause <3 s	6 (8.0)	6 (8.2)	
Pause >3 s	4 (5.3)	4 (5.5)	

bpm, beats per minute; DWP, direct wire pacing; FFR, fractional flow reserve; m, missing data; SD, standard deviation.

### Primary endpoint: Reproducibility of the FFR measurement

**Figure 3:**
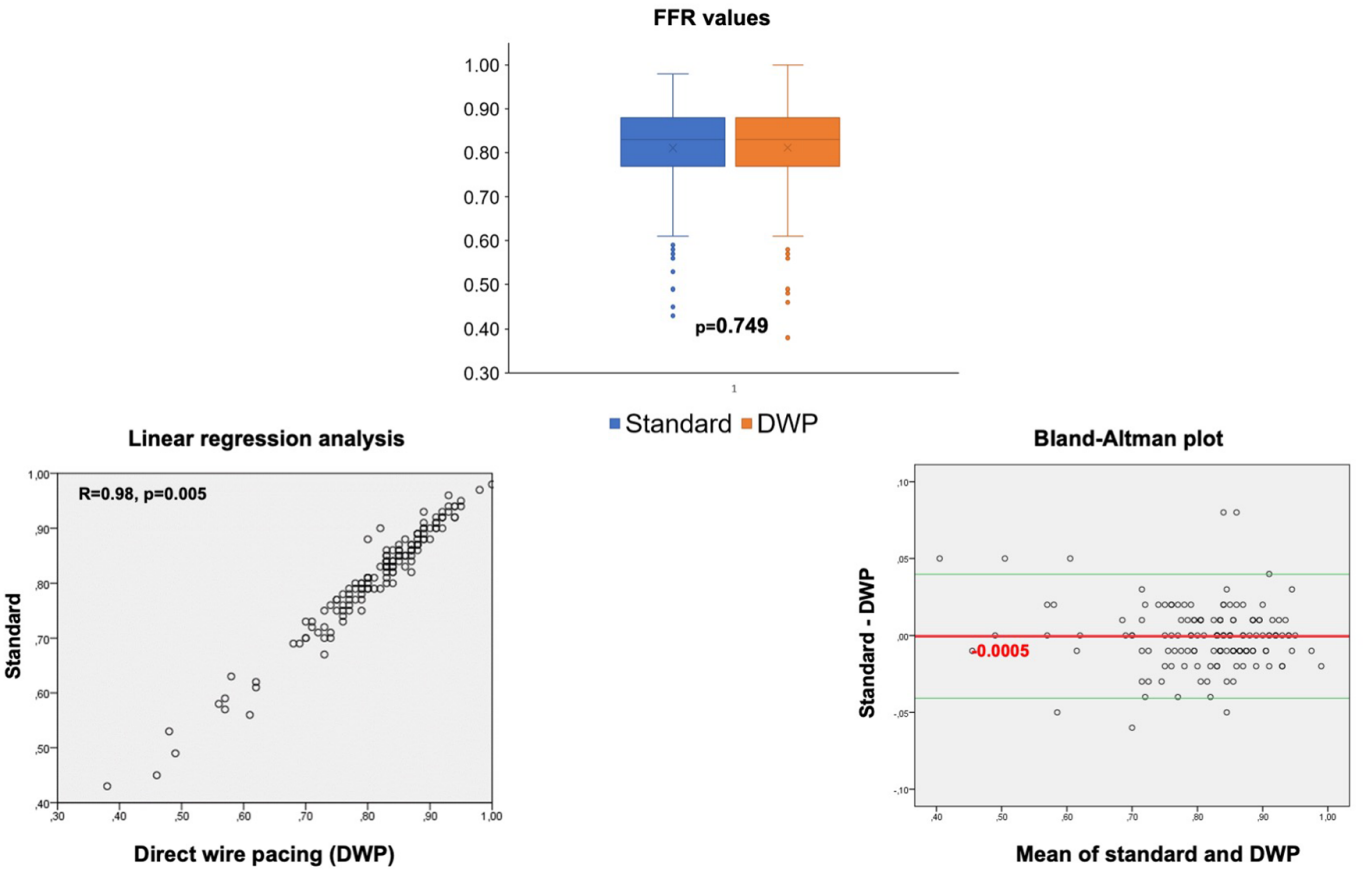
FFR values obtained with the standard method and DWP, and comparative analyses between both methods (*N* = 147).

Heart rate was significantly higher during procedures carried out with DWP stimulation than during the standard procedure (*p* < 0.001, Table 3). FFR values obtained with the standard method and with DWP were nearly identical for each lesion, with a very high degree of correlation (*R* = 0.98, *p* = 0.005) and a very high level of agreement between both methods (linear regression analysis and Bland-Altman plot) (Figure 3). The mean FFR difference of 0.00054 (95% CI: 0.004–0.003) confirmed the noninferiority of FFR measurements conducted during DWP compared to those carried out with the standard method.

**Table 3:** Heart rate and fractional flow reserve.

	Standard (*N* = 147)	DWP (*N* = 147)	*p*-value
Heart rate, mean ± SD	73 ± 10	78 ± 11	
Paired difference (standard—DWP), mean [95% CI]	−5.92 [−6.83 to −5.00]		*<0.001*
FFR, mean ± SD	0.81 ± 0.10	0.81 ± 0.10	
Paired difference (standard—DWP), mean [95% CI]	−0.00054 [−0.004 to −0.003]		*0.75*

CI, confidence interval; DWP, direct wire pacing; FFR, fractional flow reserve; SD, standard deviation.

### Secondary endpoints

#### Tolerability

**Figure 4:**
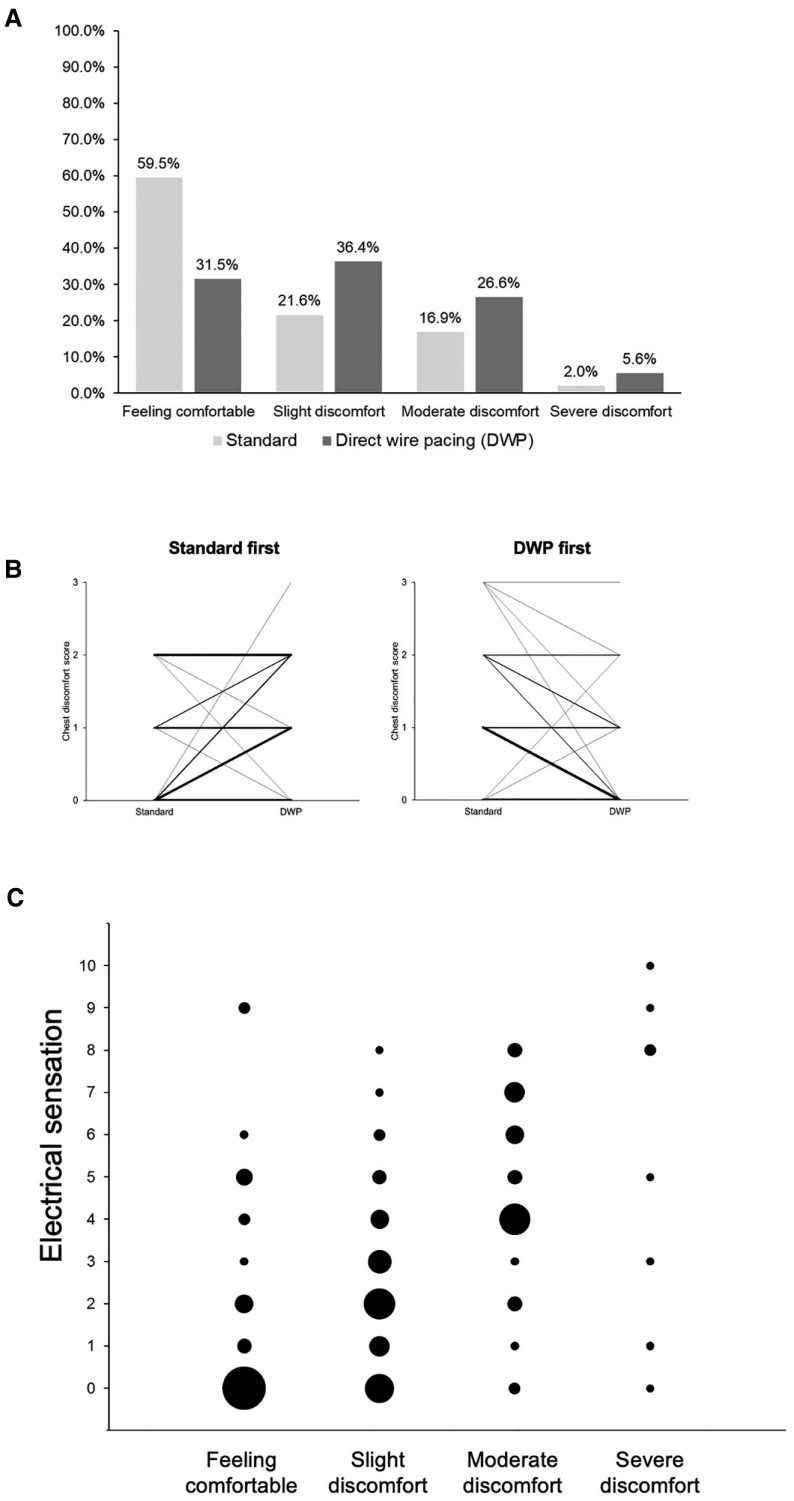
Tolerability of FFR measurements conducted using DWP and the standard method. (**A**) Chest discomfort reported after FFR measurement according to the method used. (**B**) Chest discomfort according to the intervention sequence: DWP first or standard first. Lines represent paired data. Line thickness varies according to the number of cases with the same scores: the greater the thickness, the greater the number of cases with the same score. Chest discomfort score 0 = Feeling comfortable; 1 = slight discomfort; 2 = moderate discomfort; 3 = severe discomfort. (**C**) Electrical sensations according to the degree of chest discomfort reported after FFR measurement with DWP (*p* < 0.001). Circle size varies according to the number of cases with the same scores: the larger the circle, the higher the number of cases with the same score. Electrical sensation was graded using a numeric rating scale from 0 = no pain to 10 = maximal pain. DWP: direct wire pacing.

During FFR measurement, slight to severe chest discomfort was reported in 60 cases (40.5%) after the standard method and in 98 cases (66.7%) after DWP (Figure 4A). Severe discomfort was rarely reported. The mean NRS score for the severity of chest discomfort was lower with the standard method [mean ± SD = 0.61 ± 0.84 (95% CI: 0.48–0.75) i.e., from no discomfort to slight discomfort] than with DWP [mean ± SD = 1.05 ± 0.89 (95% CI: 0.90–1.19), i.e., mild discomfort] (*p* < 0001). The mean chest discomfort difference between the two methods was significantly higher in cases who received the DWP method first than in those who received the standard method first (0.44 ± 0.70 vs. 0.4 ± 0.69, *p* < 0.001). However, chest discomfort scores did not always vary in the same direction: in some cases, lower chest discomfort was reported with the DWP method when this intervention was performed before (*n* = 31) or after (*n* = 5) the standard method and vice versa (Figure 4B).

Overall, the mean NRS score for electrical sensations reported for FFR measurements performed with DWP was 2.86 ± 2.65 (mean ± SD) out of 10. A significant relationship was observed between the occurrence of electrical sensations and the chest discomfort level reported after FFR measurements during DWP (*p* < 0.001) (Figure 4C).

#### Safety

**Figure 5:**
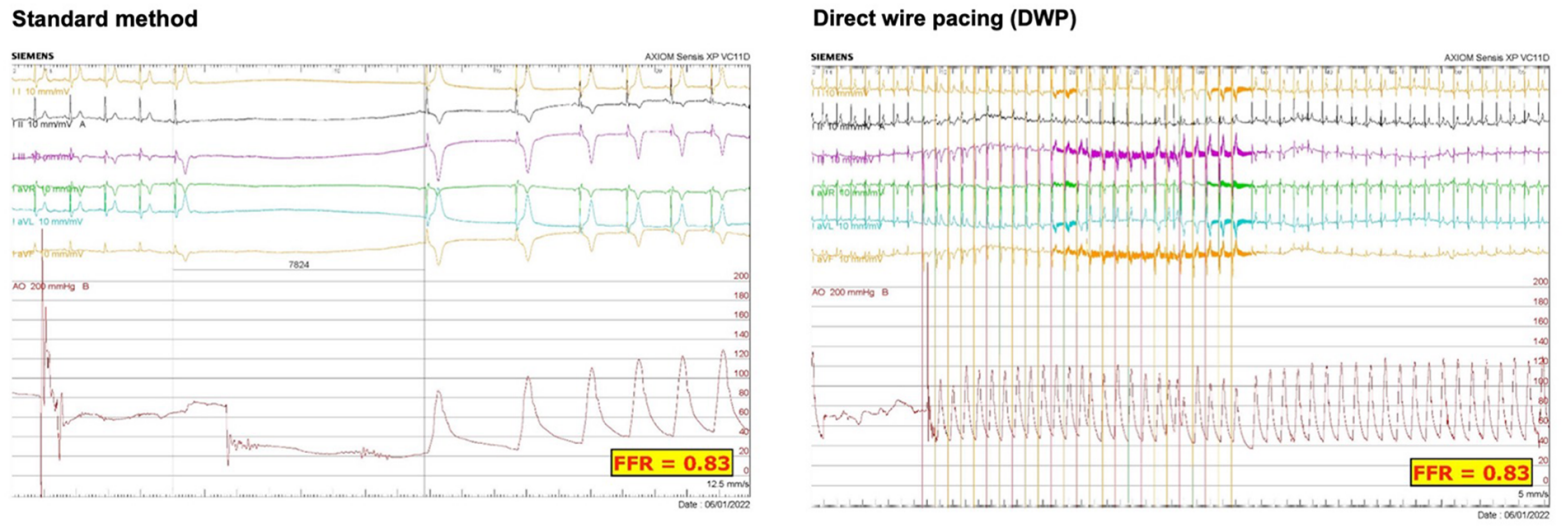
ECG monitoring recorded during the whole duration of FFR measurement with the standard method and during DWP in the same patient.

**Figure 6:**
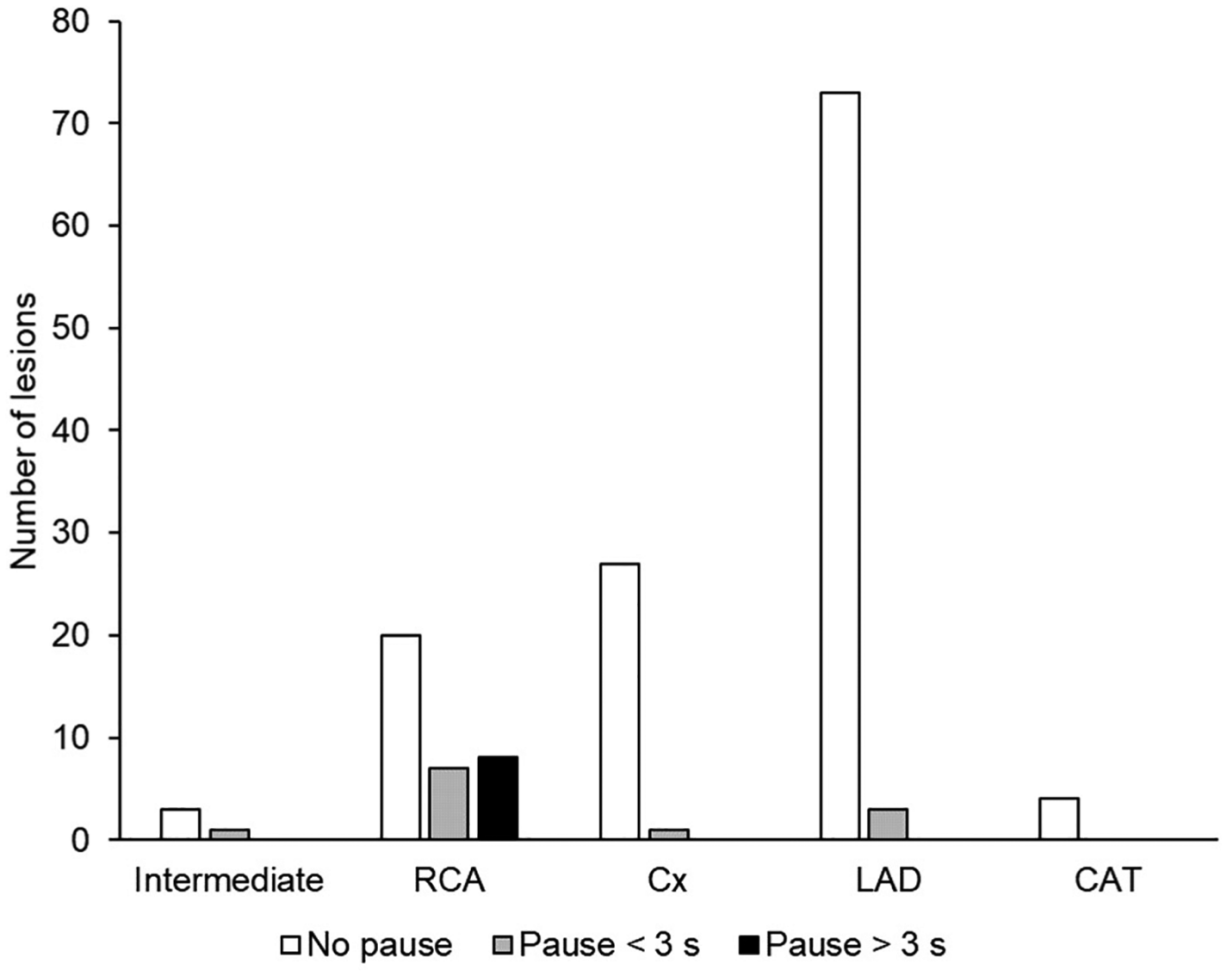
Occurrence of cardiac pauses, according to the target artery, during FFR measurement with the standard method (*N* = 147). CAT, common arterial trunk; Cx, circumflex artery; LAD, left anterior descending artery; RCA, right coronary artery.

Pauses, shown in Figure 5, were observed for 20/148 lesions (13.5%) when FFR was measured with the standard method. Most of these events (*n* = 15/20) were observed when the stenosis was in the RCA (*n* = 7 with a duration <3 s, and *n* = 8 with a duration >3 s; Figure 6). A significant correlation was observed between the target coronary artery and the occurrence of pauses in the RCA (*p* < 0.001) but there was no correlation between the occurrence of pauses and chest discomfort reported during FFR measurement with the standard method (*ρ *= 0.108, *p* = 0.19).

No pause were observed when FFR measurements were performed during DWP (Figure 3). Vagal malaise with bradycardia and hypotension, requiring the administration of atropine and ephedrine, occurred in the subject who had an occlusive coronary dissection due to FFR guidewire and guiding catheter manipulation. No other adverse events occurred.

## Discussion

To the best of our knowledge, this is the first study using DWP during FFR measurement to eliminate the drawbacks of adenosine-induced bradyarrhythmia. Our results provided evidence that FFR measurements were accurate and reliable when carried out with DWP through the metallic FFR guidewire already used in the standard procedure. The use of DWP during FFR measurement was demonstrated to be noninferior to the standard method, regardless of the target coronary artery. Moreover, no pauses were observed when DWP was used during the FFR measurements.

Coronary pressure-derived FFR was recommended for the first time in 2010 (8) and is still recommended by European guidelines (26) Nevertheless, this technique may be underused in clinical practice, partly due to the pauses/bradycardia and other side effects induced by adenosine (13). The DWP technique was originally developed to reduce complications during percutaneous coronary interventions (27, 28); single-arm studies have previously shown rapid ventricular pacing *via* the left ventricular guidewire during TAVI to be safe, effective, reliable, reproducible, and well tolerated by patients (16, 18). A multicentre randomized controlled trial (EASY TAVI) comparing the DWP technique to the standard RV-stimulation (temporary pacing catheter) in 302 patients (17) showed the superiority of the DWP technique in terms of procedural and radiation times and cost, and showed a trend toward a reduction in the occurrence of complications during TAVI. Therefore, the transfer of the use of DWP to other procedures, such as FFR measurement, is of great interest. In this study, effective stimulation was achieved with a quite low pacing threshold (around 5 mA), thanks to the uncoated pressure guidewires. The pacing rate (heart rate at rest + 10 bpm) was chosen to prevent any tachycardia and therefore drop in blood pressure (<80 mmHg), both of which may lead to an overestimation of the FFR (29, 30), and therefore to inappropriate treatment decisions. With an absolute mean difference of 6 bpm between resting and stimulated heart rates, it did not result in blood pressure drop and the FFR values obtained in our study after DWP were thus reliable. Our results showed the absence of pauses/bradycardia during FFR measurements with DWP, without any negative impact on the accuracy of the FFR results. This finding therefore supports the use of DWP during FFR measurement, especially when stenoses are in the RCA, where most pauses were observed. This could also suggest using a sentinel set-up of the external pacemaker during FFR measurements.

Some subjects in our study reported higher levels of chest discomfort during FFR measurement with DWP than during that with the standard method, although this difference was relatively small, with only few cases of severe chest discomfort. Increased chest discomfort reported during procedures with DWP correlated with increased electrical sensations experienced by the subjects, whereas pauses >3 s were not related to any chest discomfort. These findings suggest that, for patients, experiencing electrical sensations was more unpleasant than the bradycardia induced by adenosine. The development of new, purpose-built DWP devices may reduce the level of discomfort experienced by patients during DWP thanks to the absence of a mandatory needle and the use of a very low pacing threshold. Indeed, in the first-in-man study using the Electroducer Sleeve, 55/60 subjects (91.7%) reported no pain, and the mean NRS score was very low (0.13 ± 0.47 out of 10) (FIM study; Wintzer-Wehekind et al., Eurointervention, in press). Therefore, using such an innovative device will undoubtedly reduce patient discomfort during FFR measurement with DWP. In addition, using the DWP technique with a sentinel set-up could limit the electrical sensations by reducing the number of paced-beats, while avoiding the potential pauses induced by adenosine.

The validity of the FFR measurements taken during or just after a severe cardiac pause or coughing (10), as well as just after external heart massages, could be questioned. In the literature, severe pauses are little studied or reported and the real value of an FFR after a pause of 8 s for example is not known. Indeed, intense breathing can create FFR variations, external massage and coughing create artifacts and the quantities of adenosine and therefore the quality of the hyperaemia remain hypothetical and unknown after several seconds of such manoeuvres. The use of DWP in this context may serve to obtain accurate FFR values, avoiding heart pauses that are stressful for cathlab team. This technique could thus increase the use of FFR measurements for decision making in clinical practice.

## Study limitations and strengths

The main limitation of our proof-of-concept study design was that the single-centre approach may limit the generalizability of our findings. Larger-scale multicentre studies are needed to evaluate interobserver variability and allow quality control assessments of the stenosis severity determined by FFR using the DWP technique. However, FFR measurements were performed for large number of lesions (*N* = 147) by four investigators. Moreover, in this randomized crossover trial, FFR was measured sequentially for the same lesion using two methods; thus, subjects acted as their own control and between-subject variability was removed. Comparisons were made at the level of the individual rather than between groups, allowing participants to compare their experiences of the two interventions. In addition, subjects were not informed before the intervention of the order of the procedures, thus minimizing statistical bias. The 2-minute washout period between both FFR measurements eliminated any potential “carry over effect” (31).

It is also important to note that the goal of our study was to evaluate the proof-of-concept of using DWP during FFR measurement, rather than to compare the FFR measurements obtained with the degree of coronary stenosis. Thus, no quantitative coronary analysis of angiograms was performed.

Our method was innovative: the DWP technique is proving to be an asset in various cardiological procedures, by saving time, being simple to use, and improving safety by ensuring a lower proportion of adverse events. Studies evaluating the feasibility of expanding the use of the DWP technique to wider range of procedures are therefore of fundamental interest, particularly in the case of FFR measurements where our findings have shown that the use of DWP met the objective of reducing the frequency of adverse events without sacrificing the accuracy of the FFR results. The pressure guidewire used in this study was an optical fibre (OptoWire™, OpSens Medical). Further studies are needed to assess the use of DWP with piezoelectric devices.

## Conclusions

This randomized, noninferiority, crossover study showed that the use of DWP during FFR measurement resulted in accurate and reproducible FFR values, allowing pressure assessments to be conducted under maximal hyperaemia without the occurrence of the adverse events (i.e., heart pauses/bradycardia) associated with adenosine. This innovative technique could provide an alternative to using Pd/Pa and iwFR methods, allowing cardiologists to obtain more accurate FFR values to make the most appropriate treatment decisions. Although higher levels of chest discomfort were reported with DWP than with the standard method, severe pain was rarely reported, and such discomfort, correlated to electrical sensations, could be suppressed with next generation DWP devices. Together with previous studies, these results support the use of DWP in various cardiological procedures, and thus widen the range of techniques that could benefit from DWP.

## Data Availability

The raw data supporting the conclusions of this article will be made available by the authors, without undue reservation.
